# Endovascular revascularization of TASC C and D femoropopliteal occlusive disease using carbon dioxide as contrast

**DOI:** 10.1590/S1679-45082016AO3661

**Published:** 2016

**Authors:** Cynthia de Almeida Mendes, Marcelo Passos Teivelis, Sergio Kuzniec, Juliana Maria Fukuda, Nelson Wolosker

**Affiliations:** 1 Hospital Israelita Albert Einstein, São Paulo, SP, Brazil.

**Keywords:** Angioplasty, Renal insufficiency, Peripheral arterial disease, Carbon dioxide, Endovascular procedures

## Abstract

**Objective:**

To analyze the results of ten angioplasties of TASC C and D femoropopliteal lesions using CO_2_ as primary contrast in patients with no formal contraindication to iodine, aiming to decrease allergic reactions and potential nephrotoxicity in high-risk patients.

**Methods:**

We describe the results of ten angioplasties of TASC C and D femoropopliteal lesions using CO_2_ as primary contrast in patients with high risk for open revascularization and no formal contraindication to iodine. We analyzed feasibility of the procedures, complications, quality of the angiographic images, clinical and surgical outcomes, and costs of C and D lesions treated using CO_2_ as contrast medium.

**Results:**

The use of CO_2_ in C and D lesions needed iodine complementation in most of the cases (nine cases) but decreased the potential nephrotoxicity of iodine contrast medium by the reduction of its volume in this group of high-risk patients. The extension of the arterial lesions was the factor that most contributed to the need for iodine supplementation due to the difficulty to visualize the refill after a long arterial occlusion.

**Conclusion:**

The use of CO_2_ as contrast in patients with C and D lesions with no restriction for iodine contrast medium was an alternative that did not dismiss the need of iodine supplementation in most of the cases, but could decrease the potential nephrotoxicity of iodine constrast medium.

## RESUMO

**Objetivo:**

Analisar os resultados de dez angioplastias de lesões fêmoro-poplíteas TASC C e D utilizando CO_2_ como meio de contraste primário em pacientes sem restrição ao meio de contraste iodado com o objetivo de diminuir reações alérgicas e potencial de nefrotoxicidade em pacientes de alto risco.

**Métodos:**

Descrevemos os resultados de dez angioplastias de lesões fêmoro-poplíteas TASC C e D utilizando CO_2_ como meio de contraste primário em pacientes de alto risco para revascularização aberta e sem contraindicação formal a iodo. Analisamos possibilidade de execução dos procedimentos, complicações, qualidade das imagens obtidas, desfechos clínicos e cirúrgicos e custos das lesões C e D tratadas com CO_2_ como meio de contraste.

**Resultados:**

O uso de CO_2_ nas lesões C e D necessitou de complementação de iodo na maioria dos casos (nove casos), porém reduziu o potencial de nefrotoxicidade do meio de contraste iodado, diminuindo seu volume nesse grupo de pacientes de alto risco. A extensão das lesões arteriais foi o fator que mais contribuiu para necessidade de suplementação de iodo, devido à dificuldade de visualizar o reenchimento após oclusão arterial longa.

**Conclusão:**

O uso de CO_2_ como contraste em pacientes com lesões C e D sem restrição ao meio de contraste iodado foi uma alternativa que não excluiu a necessidade de suplementação com iodo na maioria dos casos, porém pôde diminuir o potencial de nefrotoxicidade do meio de contraste iodado.

## INTRODUCTION

Revascularization is the treatment of choice in cases of critical limb ischemia and it can be accomplished by open surgery or endovascular procedures, depending on the extent of lesions and clinical conditions of the patient.^(1)^ According to the 2007 Trans-Atlantic Inter-Society Consensus (TASC) II, for TASC A and B lesions, percutaneous transluminal angioplasty is the preferred treatment, and surgical bypass is recommended for good-risk patients with TASC C and D lesions.^(2)^ Many patients with TASC C and D lesions are considered to have high risk for surgical bypass due to medical comorbidities and therefore depend on endovascular techniques for revascularization. With the improvement of the technique in endovascular procedures and technological advances, currently many centers are using endovascular therapy as the primary treatment for TASC C and D lesions and reserving surgical therapy for failed endovascular intervention.^(3)^

Endovascular interventions for TASC D lesions can be safely performed with good hemodynamic improvement and limb salvage rates.^(4)^ Recent studies suggest that the “endovascular first” approach for TASC C and D is a reasonable option, with 68% patency rate at six months.^(5)^

Iodine contrast medium (ICM) is considered gold standard in endovascular procedures. However nephrotoxicity and hypersensitivity to ICM are causes for concern that limit its indiscriminate use.^(6,7)^

The use of carbon dioxide (CO_2_) is currently considered as a good alternative for aortic,^(8)^ aortoiliac,^(9)^ and femoropopliteal procedures^(10)^ in patients with formal contraindication to ICM. CO_2_ has also been used in femoropopliteal angioplasties of TASC A and B lesions in patients with no contraindication to iodine with good results.^(11)^ Specific research for the endovascular treatment of TASC C and D lesions has not yet been conducted.

## OBJECTIVE

To analyze the results of ten angioplasties of TASC C and D femoropopliteal lesions using CO_2_ as primary contrast in patients with no formal contraindication to iodine, aiming to decrease allergic reactions and potential nephrotoxicity in high-risk patients.

## METHODS

From June 2012 to September 2013, ten patients with peripheral arterial disease, arterial lesions classified as TASC C or D, and adequate runoff (identified on preoperative computed tomography angiography) underwent femoropopliteal revascularization by endovascular technique. All of them had critical limb ischemia with gangrene, and all were considered as high operative risk for surgical bypass. None of the patients exhibited severe chronic obstructive pulmonary disease. The Ethics Committee for Analysis of Research Projects on Human Experimentation approved this study at our institution, CAAE: 15117914.3.0000.0071, and all the patients signed Informed Consent Terms.

Table 1 shows the demographic and clinical profile of the patients in our study sample.

**Table 1:** Demographic and clinical profile of patients

Age average, years (SD)	79 (7.07)
Age range, years	55-78
Male gender (%)	7 (70)
BMI average, kg/m^2^ (range)	25.5 (21.5-30.7)
Hypertension (%)	9 (90)
Diabetes (%)	7 (70)
Dyslipidemia (%)	1 (10)
History of tobacco use (%)	3 (30)
Coronary disease (%)	2 (20)

SD: standard deviation; BMI: body mass index.

All procedures were performed in an endovascular suite operating (Philips Allura Xper FD, Netherlands) room under general anesthesia with cardiac monitoring, invasive arterial pressure monitoring, and bladder catheterization.

The patients underwent femoropopliteal angioplasties by the same surgical team using a constant surgical technique throughout the study. A 40-degree Trendelenburg tilt was maintained throughout the procedure.

Ipsilateral puncture of the common femoral artery was used as access in nine out of the ten patients. In one case, due to accidental loss of the ipsilateral puncture, a contralateral approach was used.

After arterial puncture and insertion of a 6F introducer access sheath, systemic heparinization (70U/kg) was performed followed by initial angiography. The lesion was crossed using catheters and hydrophilic guidewires, followed by dilatation with balloon angioplasty and new control angiography to evaluate the need for stenting. At the end of the procedure, heparinization was reversed with protamine, and local manual compression was carried out for 30 minutes after removal of the introducer.

Immediately after the operation, on a routine basis in the service, all patients were referred to the Intensive Care Unit for at least 24 hours, and received intravenous fluids following a fixed protocol for renal protection. Thereafter patients remained hospitalized for the time needed, with daily monitoring of renal function, blood count, and electrolytes for at least 72 hours.

The CO_2_ injection was performed manually, without any specific pump system. A cylinder with medicinal CO_2_, a particle filter (Millex^®^ Durapore^®^ hydrophilic 0.22μm pore), and a three-way stopcock were used for aspiration of CO_2_ and the entire procedure was held inside a bowl with saline solution. After capturing the required volume of CO_2_, additional 3 to 5mL of saline solution were aspired into the syringe to provide a water seal while the tip was kept down. Thus, a physical barrier was created between the room air and the CO_2_ content, which is independent of manual compression and is safe of air contamination.^(12)^ We used 10mL and 20mL syringes for the intra-arterial contrast injection, performed into the femoral introducer or through an end hole catheter.

The injection of ICM, when needed, was performed manually in 10mL syringes using 3mL of iodinated contrast media and 7mL of saline solution per injection.

The endovascular material (sheaths, guidewires, and stents) used in all procedures was provided by Cook Medical Inc. The material used in each intervention and the volumes of CO_2_ and ICM were precisely recorded for further analysis.

All the procedures were recorded on DVD for further analysis by two observers who did not take part in the intervention and who had no experience with the use of CO_2_. Both observers analyzed each film separately.

Observers attributed a score for each evaluated image ranging from 1 to 3. The score 1, considered poor, was attributed when there was significant loss of definition in the vessels and/or collateral circulation which precluded the procedure; the score 2, considered fair, was attributed when there was some loss of definition in the vessels and/or collateral circulation, but did no hinder intervention; the score 3, considered good, was assigned when there was good contrast in the vessels and collateral circulation.

An individual analysis was carried out for each intervention, evaluating costs related to the contrast media and endovascular material used (puncture needles, sheaths, angioplasty balloons, catheters, insufflating syringes, and stents). It is relevant to emphasize that, since ICM is required for balloon filling during angioplasties, we added the price of one 20mL flask of ICM to all the patients who demanded no or less than 20mL of iodinated contrast.

We evaluated the following outcomes: feasibility of the procedures; complications; surgical outcomes (ankle-brachial index − ABI variation, frequency of major amputation in 30 days); creatinine clearance rate using the Cockcroft-Gault formula;^(13)^ quality of the angiographic images obtained with CO_2_; costs of the endovascular materials; and costs of contrast agents.

### Statistical analysis

Categorical variables were described as absolute frequencies and percentages. The distribution of numerical variables was investigated by histograms and normality Shapiro-Wilk tests. Numerical data were described by means and standard deviations (SD) or medians and interquartile ranges (first quartile to third quartile) in the case of non-normally distributed data.

Statistical analysis was performed using Statistical Package for Social Science (SPSS) released in 2008, for Windows, version 17.0. The level of significance used was 5%.

## RESULTS

Nine patients had TASC D lesions, and one patient had a TASC C lesion. All of them had critical limb ischemia with gangrene, and all were considered as high operative risk for surgical bypass.

There were no CO_2_ related complications. The injected volume of CO_2_ required for each procedure ranged from 12 to 146mL (mean of 70.35mL; SD of 40.5).

In nine patients (one TASC C and eight TASC D), the use of iodine was necessary for completion of the procedure. In these cases, the iodine use was justified by difficulty in visualizing refill after occlusion. The mean volume of ICM used per patient was 11.9mL, ranging from 5 to 23mL (mean of 11.9mL; SD of 7.7). In five cases, the volume of ICM used was inferior to 10mL, and in the other four cases, less than 25mL.

We were able to perform the proposed procedures, with successful target vessel revascularization, in six of ten patients treated in this series.

All the patients in whom the procedure could not be completed had TASC D lesions. In two of them, we could not accomplish the guidewire re-entry into the arterial tree after the occluded segment, and we did not have re-entry devices in our service. In one case, there was no adequate runoff, despite preoperative computed tomography angiography suggesting otherwise.

In the last unsuccessful case, there was a slow contrast flow in the distal arterial bed, probably by distal embolization. This patient underwent femoropedal bypass in postoperative day one, but this procedure was not successful as well, and major amputation (above the knee) was performed within 30 days of intervention.

Two out of the ten patients presented with acute myocardial infarction and one of them died as a result of cardiac complications, occurring within 30 days of intervention.

The duration of the procedures did not exceed three hours in neither of the patients.

Operative details, including costs of endovascular material, contrast costs, fluoroscopy time, duration of the procedures, variations in creatinine clearance levels, and ABI of all patients are shown in tables 2 and 3.

**Table 2:** Operative details

Patient	TASC classification	Preoperative creatinine clearance	Postoperative creatinine clearance	Preoperative ABI	Postoperative ABI	Duration of surgery (minutes)
1	D	25.86	28.95	0	0.2	80
2	D	40.37	74.53	0.2	0.33	180
3	D	45.69	55.57	0.1	0.2	120
4	D	73.35	87.71	0.5	0.6	130
5	D	28.65	42.89	0.4	0.45	130
6	D	29.85	36.55	0.57	0.21	105
7	D	80	101.49	0	0	180
8	D	60.1	38.89	0.47	0.87	75
9	D	52.5	44.44	0	0.56	75
10	C	110.38	100.34	0.25	0.6	100

TASC: Trans-Atlantic Inter-Society Consensus; ABI: ankle-brachial index.

**Table 3:** Operative details

Patient	CO_2_ volume (mL)	ICM volume (mL)	Endovascular material cost (US Dollar)	Contrast cost (US Dollar)	Technical success
1	45	23	1,472	20.12	No
2	58	9	3,002	10.12	Yes
3	41	9	8,417	10.12	Yes
4	78	9	9,042	10.12	Yes
5	56	0	7,362	10.12	Yes
6	85	15	1,667	10.12	No
7	146	7	23,112	10.12	No
8	127.5	5	15,142	10.12	Yes
9	55	21	3,442	20.12	No
10	12	21	5,372	20.12	Yes

CO_2_: carbon dioxide; ICM: iodine contrast medium.

The evaluation of angiographies by the two observers is presented in table 4.

**Table 4:** Evaluation of angiographies by observers

Observer	CO_2_ arteriography		

	Good	Fair	Poor
1	4	4	2
2	3	7	0

CO_2_: carbon dioxide.

CO_2_ arteriograms were graded as good or fair by both observers with high interobserver image quality agreement. Only two images were graded as poor.

**Figure 1:**
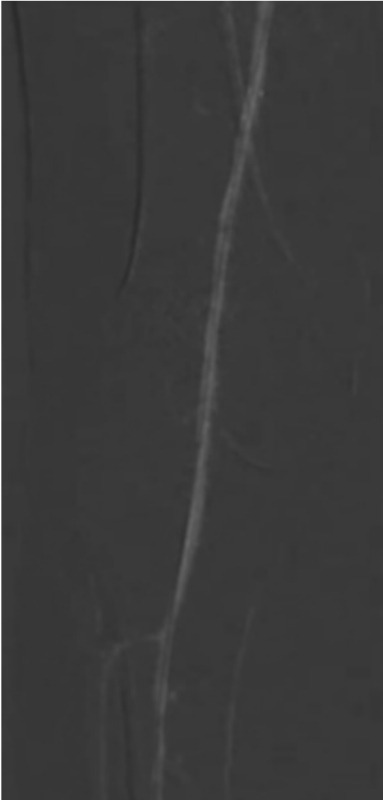
Example of a carbon dioxide arteriography

Additionally, after six months of follow-up, two other patients underwent major amputation (one in which we did not have initial technical success and was deemed as high-risk for open revascularization, and the other one that suffered occlusion of the stent and was as well deemed as high-risk for surgical revascularization). The remaining seven patients were alive and free of amputation. Figure 1 illustrates a CO_2_ arteriography.

## DISCUSSION

Endovascular surgery has an increasingly important place in the revascularization of the lower limbs. The “endovascular first” approach is already a reality in many centers. Patients with severe chronic arterial occlusive disease and extensive lesions classified as TASC C and D have the chance of limb salvage attempt with endovascular surgery. These are complex patients with extensive arterial lesions and often with very serious clinical comorbidities, making them at high risk for open limb revascularization. Frequently the endovascular surgery is the only option for limb salvage attempt.

In this series of cases, we simultaneously had technical difficulty of an endovascular approach of TASC C and D lesions and the attempt to do this procedure with an intravascular contrast different than the usual. Our group already had the experience with the use of CO_2_ in TASC A and B lesions with good results. Therefore aiming to investigate the possibility of a wider use for CO_2_, we sought to establish the effectiveness of CO_2_ in patients with femoropopliteal TASC C and D lesions, initially in patients who presented with no contraindication to ICM, as if the complementation with iodine was necessary, it would not be problematic.

Our results showed that the use of CO_2_ in TASC C and D lesions needed iodine complementation in most of the cases (nine out of ten). The extension of the arterial lesions was the factor that most contributed to the need for iodine supplementation due to the difficulty to visualize the refill after a long arterial occlusion.

Although there are reports of complications with the use of intra-arterial CO_2_,^(14)^ in our series no adverse events that could be related to this particular contrast media were observed, corroborating with other larger series previously published.^(15)^ The use of CO_2_ manual injection without the need for pumps or injection systems is considered safe^(16)^ and makes its use more affordable and easily reproducible.

In this series we observed that in the cases that we could not achieve technical success, this fact was related to the anatomy of the arterial lesions and technical difficulties, rather than to the type of contrast, as even with the iodine complementation we could not achieve technical success in four cases.

Despite the fact that the observer’s evaluation had a greater amount of good/fair notes, during the intraoperative period, the surgical team decided to use iodine supplementation in most of the cases to finish the procedures satisfactorily. Perhaps with the gain of experience in these complex cases, the procedures could be finished without iodine supplementation, as well as with the use of CO_2_ in TASC A and TASC B lesions.

In previous studies of our group, the gain of experience with the use of CO_2_ enabled us to understand that the injection of CO_2_ through catheters positioned more distally in the arteries, closer to the target lesion, allowed acquisition of superior quality images, but in TASC C and D lesions this technique did not dismiss the supplementation with ICM in most of the cases.

The scores attributed by both observers constitute a descriptive approach rather than an analytical approach and therefore are a limitation of our study.

In our study, seven patients showed an increase in creatinine clearance values in the postoperative period compared to the preoperative values. This improvement in renal function may be attributed to Intensive Care Unit support and postoperative fluid therapy, but some authors suggest that CO_2_ may have a renal protective effect. Criado et al.^(8)^ reported that CO_2_ reduced contrast-induced nephropathy in patients who underwent endovascular repair of abdominal aortic aneurysm. Future studies with larger sample sizes are required to confirm the protective renal effect of CO_2_, even in patients with normal renal function.

We believe that CO_2_ can be an alternative in the treatment of TASC C and D lesions, because although the supplementation with iodine is commonly necessary and even expected, the burden of nephrotoxicity may be diminished, as its volume could be lowered. Aiming to decrease allergic reactions and potential nephrotoxicity in high-risk patients, the use of CO_2_ should be stimulated. The CO_2_ technique should work as a therapeutic weapon for vascular surgeons so they have the possibility of choosing the best contrast option for each patient at each moment of the procedures, instead of being limited to the use of only one type of contrast.

## CONCLUSION

The use of CO_2_ in patients with TASC C and D lesions with no restriction for iodine contrast medium was an alternative that did not dismiss the need of iodine supplementation in most of the cases, but could decrease the potential nephrotoxicity of iodine contrast medium by the reduction of its volume in this group of high-risk patients for open revascularization surgery that continues to be a challenge for endovascular surgeons.
